# Exploring the Link Between Telomeres and Mitochondria: Mechanisms and Implications in Different Cell Types

**DOI:** 10.3390/ijms26030993

**Published:** 2025-01-24

**Authors:** Graziana Assalve, Paola Lunetti, Maria Santa Rocca, Ilaria Cosci, Andrea Di Nisio, Alberto Ferlin, Vincenzo Zara, Alessandra Ferramosca

**Affiliations:** 1 Department of Experimental Medicine, University of Salento, I-73100 Lecce, Italy; graziana.assalve@unisalento.it (G.A.); paola.lunetti@unisalento.it (P.L.); vincenzo.zara@unisalento.it (V.Z.); 2 Unit of Andrology and Reproductive Medicine, University Hospital of Padova, I-35128 Padova, Italy; mariasanta.rocca@aopd.veneto.it (M.S.R.); alberto.ferlin@unipd.it (A.F.); 3 Department of Medicine, University of Padova, I-35128 Padova, Italy; ilaria.cosci@unipd.it; 4 Department of Wellbeing, Nutrition and Sport, Pegaso Telematic University, Centro Direzionale Isola F2, I-80143 Naples, Italy; andrea.dinisio@unipegaso.it

**Keywords:** telomere, telomerase, mitochondria, reactive oxygen species (ROS), telomere length

## Abstract

Telomeres protect chromosome ends from damage, but they shorten with each cell division due to the limitations of DNA replication and are further affected by oxidative stress. This shortening is a key feature of aging, and telomerase, an enzyme that extends telomeres, helps mitigate this process. Aging is also associated with mitochondrial dysfunction, leading to increased reactive oxygen species (ROS) that exacerbate cellular damage and promote apoptosis. Elevated ROS levels can damage telomeres by oxidizing guanine and disrupting their regulation. Conversely, telomere damage impacts mitochondrial function, and activation of telomerase has been shown to reverse this decline. A critical link between telomere shortening and mitochondrial dysfunction is the DNA damage response, which activates the tumor suppressor protein p53, resulting in reduced mitochondrial biogenesis and metabolic disruptions. This highlights the bidirectional relationship between telomere maintenance and mitochondrial function. This review explores the complex interactions between telomeres and mitochondria across various cell types, from fibroblasts to sperm cells, shedding light on the interconnected mechanisms underlying aging and cellular function.

## 1. Introduction

In eukaryotes, the linear structure of chromosomes necessitates a mechanism to protect DNA ends from accidental breaks. This process is crucial to prevent undesired recombination, degradation by nucleases, and inappropriate cell cycle arrest. The stability of chromosome ends is ensured by telomeres, which cap the ends of chromosomes [1]. Telomeric DNA consists of hexameric (TTAGGG) repeats organized into a T-loop structure. This structure is critical for telomere stability and is formed with the assistance of the shelterin complex, a protein hetero-hexamer comprising telomeric repeat binding factors (TRF1 and TRF2), TERF1-interacting nuclear factor 2 (TIN2), protection of telomeres protein 1 (POT1), adrenocortical dysplasia protein homolog (TPP1), and repressor activator protein 1 (RAP1) [2]. Although telomeric regions do not encode proteins, they transcribe long non-coding RNAs known as telomeric repeat-containing RNA (TERRA), which regulate telomeric chromatin structure [3].

Due to limitations in the DNA replication machinery, the synthesis of the very ends of linear chromosomes cannot be completed, leading to progressive telomere shortening with each cell division. Additional shortening occurs due to oxidative damage and other end-processing events, even in non-dividing cells [4]. Telomere attrition is a hallmark of normal aging, and, at the same time, pathological telomere dysfunction accelerates aging [5]. To counteract this inevitable shortening, several compensatory mechanisms exist. One of these mechanisms involves telomerase, a specialized reverse transcriptase that uses an RNA template to extend the G-rich strand of telomeric DNA at the 3′ overhang. Telomerase expression is notably higher in germ cells, stem cells, and progenitor cells compared to differentiated somatic cells. However, the levels of telomerase in adult somatic stem and progenitor cells are generally insufficient to fully replenish telomeric DNA during cell division, tissue regeneration, or responses to external stimuli [2].

Telomerase consists of two main components: the telomerase reverse transcriptase (TERT) and the telomeric RNA component (TERC). TERC is widely expressed, while the TERT gene is strongly repressed in most human somatic cells [6]. Hence, the factor that sets a rate limit for telomerase activity regulation is TERT. The TERT promoter region features an enhancer box (E-box), which serves as a binding site for various transcriptional activators and suppressors, along with GC-boxes that interact with zinc finger transcription factors to enhance TERT transcription [7]. Among these, cellular myelocytomatosis oncogene (c-MYC) is a transcriptional activator that directly binds to the E-box in the TERT promoter, inducing its transcription [8]. Additionally, forkhead-box-protein O3 (FOXO3a) protein modulates c-MYC activity through multiple pathways, enabling the activation of TERT transcription in a manner dependent on the c-MYC/E-box interaction [9].

Interestingly, the subunits of telomerase, TERT and TERC, also perform non-telomeric functions, particularly in regulating mitochondrial metabolism. Notably, about 10–20% of the human TERT (hTERT) protein is localized within mitochondria [4]. This localization is attributed to mitochondrial targeting sequences present at the N-terminal region of TERT, enabling its translocation to mitochondria under mild oxidative stress, mitigating DNA damage, apoptosis, and oxidative stress [10]. In contrast, high levels of oxidative stress result in a decrease in TERT expression in both nuclei and mitochondria, accompanied by reduced mitochondrial membrane potential, increased cellular reactive oxygen species (ROS) and apoptosis [11]. Additionally, the TERC can be brought into the mitochondria, where it is converted to TERC-53 and subsequently released back into the cytosol [12]. It is hypothesized that processed TERC acts as a signaling molecule, conveying information about mitochondrial abnormalities to the nucleus [10].

This review explores the complex bidirectional relationship between telomere maintenance and mitochondrial function across various cell types, with a particular focus on the distinct dynamics observed in sperm cells.

## 2. Effects of Mitochondrial Alterations on Telomere Length

A specific redox state is necessary for optimal telomerase activity, suggesting its regulation by ROS [13]. ROS modulate telomerase activity via the AKT signaling pathway, influencing the phosphorylation of the TERT subunit or through transcriptional and post-translational mechanisms, such as the translocation of TERT from the nucleus to mitochondria [11]. TERT plays a protective role in mitochondria by reducing ROS production, minimizing damage, and supporting normal mitochondrial function [14]. This underscores the integral role of TERT in mitochondrial metabolism.

During the natural aging process, mitochondrial biogenesis and bioenergetic efficiency significantly decline. Aging cells exhibit reduced mitochondrial DNA (mtDNA) content and increased ROS production, resulting in increased electron leakage, impaired cellular respiration, and reduced adenosine triphosphate (ATP) generation [15]. This progressive dysfunction heightens ROS generation, which exacerbates mitochondrial damage, induces global cellular harm, and triggers apoptosis. When ROS levels surpass a critical threshold, they shift from being protective to aggravating age-related damage [16]. The telomeric TTAGGG repeats are particularly susceptible to oxidative stress, as it causes oxidation of the free nucleotide pool, replication fork arrest, disruption of the proteins regulating telomere length, and the base modification of guanine to 8-hydroxy-2′-deoxyguanosine (8-OHdG) due to its low redox potential [17,18,19].

This connection between mitochondrial dysfunction and telomere attrition was first established experimentally using drugs that damage mitochondria. Mouse embryos treated with drugs that elevate mitochondrial ROS production exhibited telomere shortening, telomere fusions, and apoptosis [4]. In these experiments, antioxidant treatments prevented telomeric damage, confirming that mitochondrial ROS play a causal role in telomere instability. Similarly, menadione, a compound that disrupts mitochondrial complex I and promotes ROS generation, was shown to cause telomeric single-strand breaks in cultured cancer cells [20].

Mitochondrial dysfunction contributes to aging through mechanisms that extend beyond ROS production. One such mechanism involves mtDNA depletion, which has been shown to elevate the expression of heterogeneous nuclear ribonucleoproteins (hnRNPA), contributing to telomere shortening [21]. Moreover, mitochondrial dysfunction induces telomere attrition through the formation of telomere dysfunction-induced foci (TIFs). TIFs are sites at telomeres where DNA damage response (DDR) factors accumulate, signaling critically short or uncapped telomeres [22].

## 3. Effects of Telomere Shortening on Mitochondrial Alterations

Telomere shortening is strongly associated with mitochondrial dysfunction, which includes impaired mitochondrial biogenesis, reduced mtDNA replication, disrupted mitochondrial dynamics, and reprogramming of cellular metabolism [23].

### 3.1. Effects of Telomere Shortening on Mitochondrial Biogenesis and Dynamics

Studies reveal that telomeric DNA damage often precedes mitochondrial metabolic disorders, with patients suffering from mitochondrial diseases displaying shorter telomeres than healthy individuals [10]. Consequently, mitochondrial dysfunction has been linked to telomere attrition, and telomerase activation has been shown to reverse this decline [14]. As mentioned above, TERT plays a crucial role in maintaining mitochondrial integrity. It can translocate to mitochondria, where it reduces ROS production and thereby protects telomere function [24].

The effects of telomere damage on mitochondrial function have been extensively studied using telomerase-deficient mouse models [14,25]. In these mice, the absence of telomerase results in shortened telomeres, leading to mitochondrial dysfunction. In fact, mitochondrial electron transport chain activity is impaired, and ATP levels in various tissues are reduced [25]. As demonstrated in mouse studies, short telomeres have also been shown to substantially impair insulin secretion by pancreatic β-cells. This impairment in insulin release is closely associated with mitochondrial dysfunction, as islets with shortened telomeres show reduced membrane hyperpolarization, reflecting a defect in the respiratory chain [26]. Remarkably, restoring telomerase or engineering mice with extraordinarily long telomeres can protect against these phenotypes [27]. Furthermore, findings from yeast models indicate that telomere dysfunction can compromise mitochondrial function and genomic stability by impairing the biogenesis of iron–sulfur clusters, which are crucial for the proper assembly of respiratory chain complexes [28].

**Figure 1:**
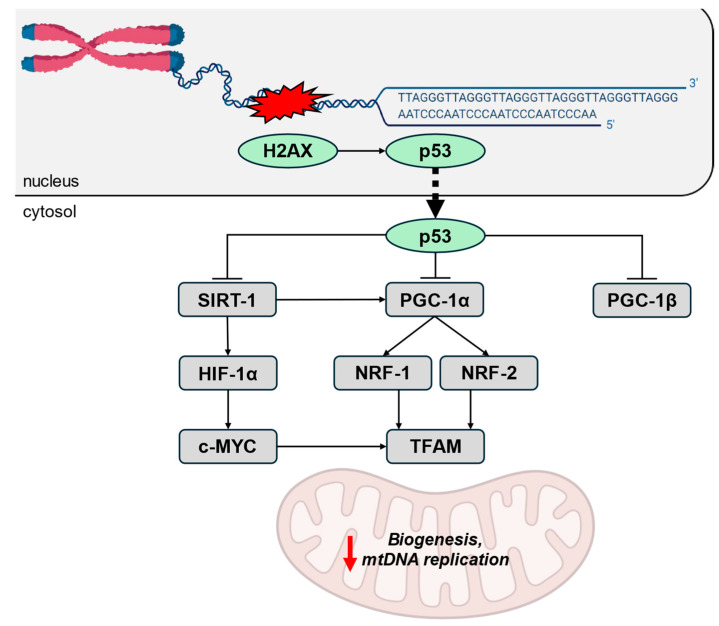
Effects of telomere damage on mitochondrial biogenesis. Telomere attrition activates H2AX, which subsequently triggers p53. In response to DNA damage signals, p53 represses the expression of SIRT1, PGC-1α, and PGC-1β. This repression ultimately leads to a decrease in TFAM level, which is directly associated with reduced mitochondrial biogenesis and impaired mtDNA replication. Activated factors are shown in green, and repressed ones in gray. Blunt arrows (┴) indicate inhibition while sharp arrows (→) indicate stimulation. c-MYC, cellular myelocytomatosis oncogene; H2AX, H2A histone family member X; HIF-1α, hypoxia-inducible factor-1α; mtDNA, mitochondrial DNA; NRF, nuclear transcriptional respiratory factor; PGC, peroxisome proliferator-activated receptor-γ coactivator; SIRT, sirtuin; TFAM, mitochondrial transcription factor A.

Hence, telomere damage appears to drive mitochondrial dysfunction through multiple signaling pathways. One proposed mechanism linking short telomeres to impaired mitochondrial function involves the DDR and leads to the reprogramming of mitochondrial biogenesis. Telomere attrition activates DDR proteins, such as H2A histone family member X (H2AX), which subsequently trigger the tumor suppressor gene p53 [27]. As a key regulator of the cell cycle and a DNA damage sensor, p53 plays a central role in maintaining cellular homeostasis and genetic stability. Upon telomere attrition, p53 responds to DNA damage signals by repressing the expression of the peroxisome proliferator-activated receptor-γ coactivator (PGC)-1α and PGC-1β, two master regulators of mitochondrial biogenesis [4,29]. This suppression leads to reduced mitochondrial biogenesis [30,31]. A decreased mtDNA/nuclear DNA ratio has been reported to be associated with impaired mitochondrial biogenesis, as mtDNA replication is regulated by the PGC-1 protein family [23]. In particular, PGC-1α induces the nuclear transcriptional respiratory factor (NRF)-1 and NRF-2, which control the mitochondrial transcription factor A (TFAM) that is necessary for mtDNA maintenance [32]. p53 activation also results in decreased expression of sirtuin 1 (SIRT1), a nicotinamide adenine dinucleotide (NAD)-dependent deacetylase involved in the deacetylation of p53 and activation of PGC-1α. Together, SIRT1 and PGC-1α regulate the DDR and mitochondrial homeostasis [33]. By limiting SIRT1 activity, p53 activation further exacerbates mitochondrial dysfunction [34]. Experimental interventions, such as forced expression of PGC-1α or deletion of p53 in the context of telomere dysfunction, have been shown to restore mitochondrial respiration [10]. In addition to the SIRT1-PGC-1α axis, SIRT1 has also been proposed to regulate TFAM via the SIRT1-HIF-1α-c-MYC pathway, independently of PGC-1α [35]. Hence, the downregulation of SIRT1 is also associated with a decrease in TFAM levels, which correlates with reduced mitochondrial biogenesis. Interestingly, calorie restriction has been found to prevent the age-related declines in TFAM level, suggesting that metabolic interventions can influence the relationship between telomere length, TFAM expression, and mitochondrial biogenesis (Figure 1) [36].

**Figure 2:**
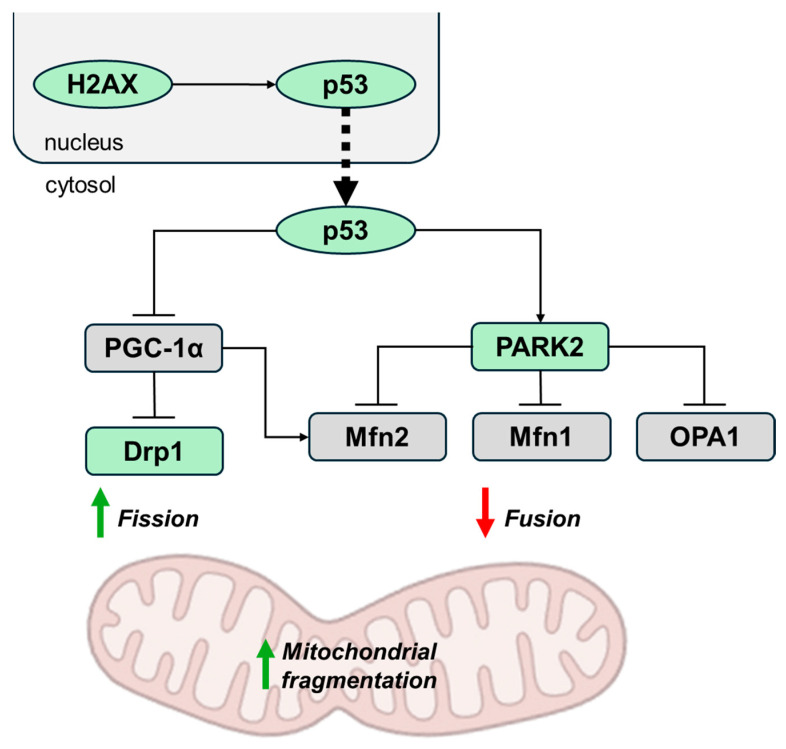
Effects of telomere damage on mitochondrial dynamics. Telomere attrition-induced activation of p53 leads to repression of PGC-1α and the activation of PARK2, resulting in increased mitochondrial fragmentation. The repression of PGC-1α disrupts the balance between mitochondrial fusion and fission by reducing the expression of the fusion protein Mfn2 and impairing the inhibition of the fission protein Drp1. At the same time, the activation of PARK2 promotes the ubiquitination and subsequent degradation of the mitochondrial fusion proteins Mfn1, Mfn2, and OPA1. Activated factors are shown in green, and repressed ones in gray. Blunt arrows (┴) indicate inhibition while sharp arrows (→) indicate stimulation. Drp1, dynamin-related protein 1; Mfn, mitofusin; OPA1, optic atrophy 1.

Disruptions in mitochondrial dynamics are also linked to the age-related decline in mitochondrial biogenesis. These dynamics involve the movement of mitochondria along the cytoskeleton, as well as the regulation of their architecture and connectivity through coordinated fusion and fission events. Maintaining a proper balance between fusion and fission is crucial for ensuring mitochondrial quality and integrity, and any imbalance in these processes can lead to the accumulation of damaged mitochondria [37]. Mitochondrial fusion and fission are orchestrated by a variety of proteins. Mitofusins (Mfn1 and Mfn2) are critical for the fusion of the outer mitochondrial membrane, while optic atrophy 1 (OPA1) is primarily involved in the fusion of the inner mitochondrial membrane. For mitochondrial fission, dynamin-related protein 1 (Drp1) plays a pivotal role. Drp1 is recruited to the mitochondrial outer membrane by specific proteins, including mitochondrial fission 1 (Fis1), mitochondrial dynamics proteins of 49 and 51 kDa (MiD49/51), and mitochondrial fission factor (Mff). These proteins form fission sites where Drp1 assembles into higher-order spiral complexes, constricting mitochondria to enable division [38]. PGC-1α plays a dual role in mitochondrial dynamics by promoting fusion and suppressing fission. It achieves this balance by upregulating Mfn2 expression and inhibiting Drp1 activity [39]. Consequently, telomere attrition-induced repression of PGC-1α results in increased mitochondrial fragmentation. Additionally, mitochondrial fusion proteins can be ubiquitinated by the E3 ligase Parkin (PARK2), which is activated by p53 (Figure 2). This ubiquitination targets them for degradation by proteases, ultimately reducing mitochondrial fusion and promoting the autophagic degradation of mitochondrial organelles [40].

### 3.2. Effects of Telomere Shortening on Mitochondrial Metabolism

Reduced mitochondrial biogenesis is closely linked to other dysfunctions, including metabolic impairments that significantly diminish ATP synthesis capacity. Consequently, telomere dysfunction leads to the accumulation of diverse extracellular metabolites collectively referred to as the “extracellular senescence metabolome” (ESM) [41]. Knock-out studies of shelterin subunits reveal that all, except TRF1, are involved in mitochondrial metabolism [42]. However, it is important to note that telomere metabolism may differ between species, such as human cells and knock-out mice, which could limit direct comparisons. Notably, the glycolysis and pentose phosphate pathway (PPP) are modulated when shelterin components are knocked out, indicating that telomere dysfunction can reprogram cellular metabolism to optimize energy usage under stress. TIN2, in particular, localizes to mitochondria and modulates their activity and ROS production [43]. TIN2-specific knock-out disrupts the tricarboxylic acid (TCA) cycle metabolites and downregulates SIRT3, leading to mitochondrial dysfunction [44].

**Figure 3:**
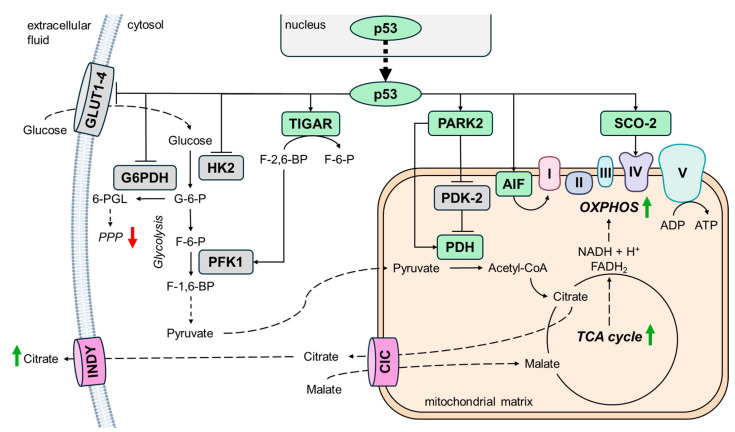
Effects of telomere damage on cellular metabolism. Telomere attrition activates p53, which suppresses glycolysis and PPP in favor of mitochondrial respiration. Specifically, p53 inhibits glycolysis by reducing the expression of glucose transporters GLUT1 and GLUT4, downregulating HK2, and activating TIGAR. In parallel, p53 suppresses the PPP by reducing G6PDH activity. Simultaneously, p53 promotes mitochondrial OXPHOS through two mechanisms. First, it inhibits PDK2 and upregulates PDH via PARK2 activation. Second, it enhances the expression of AIF and SCO2, which are crucial for the assembly and functionality of mitochondrial respiratory complexes. These metabolic alterations may lead to the accumulation of TCA cycle metabolites, such as citrate, which is then transported to the cytosol by the mitochondrial carrier CIC and subsequently exported into the extracellular fluid by plasma membrane transporters. Activated factors are shown in green, and repressed ones in gray. Blunt arrows (┴) indicate inhibition while sharp arrows (→) indicate stimulation. The 6-PGL, 6-phosphogluconolactone; ADP, adenosine diphosphate; AIF, apoptosis-inducing factor; ATP, adenosine triphosphate; CIC, mitochondrial citrate carrier; F-1,6-BP, fructose 1,6-bisphosphate; F-2,6-BP, fructose 2,6-bisphosphate; F-6-P, fructose-6-phosphate; FADH_2_, flavin adenine dinucleotide; G-6-P, glucose-6-phosphate; GLUT, glucose transporter; HK2, hexokinase 2; NADH, nicotinamide adenine dinucleotide; OXPHOS, oxidative phosphorylation; PDH, pyruvate dehydrogenase; PDK2, pyruvate dehydrogenase kinase 2; PFK1, phosphofructokinase 1; PPP, pentose phosphate pathway; SCO2, synthesis of cytochrome c oxidase 2; TCA, tricarboxylic acid; TIGAR, TP53-inducible glycolysis and apoptosis regulator.

Additionally, the activation of p53 mediated by telomere attrition induces alterations in cellular metabolism. Indeed, p53 limits glucose uptake by reducing the expression of glucose transporters GLUT1 and GLUT4. p53 also regulates glucose metabolism through direct or indirect regulation of the expression of key enzymes involved in glycolysis [45]. For instance, p53 downregulates protein levels of hexokinase 2 (HK2) and it transcriptionally activates the TP53-inducible glycolysis and apoptosis regulator (TIGAR). TIGAR inhibits glycolysis at an early stage by lowering fructose-2,6-bisphosphate levels, a metabolite that normally allosterically activates phosphofructokinase 1 (PFK1). p53 also binds and suppresses glucose-6-phosphate dehydrogenase (G6PDH), reducing PPP activity. Furthermore, p53 promotes mitochondrial oxidative phosphorylation (OXPHOS) by inhibiting pyruvate dehydrogenase kinase 2 (PDK2) through PARK2 activation [28,41]. PDK2 is a negative regulator of pyruvate dehydrogenase (PDH) complex that converts pyruvate to acetyl-CoA, a primary substrate for the TCA cycle. Thus, the inhibition of PDK2 by p53 activates PDH, which in turn promotes mitochondrial OXPHOS. Additionally, PARK2 increases the protein expression of pyruvate dehydrogenase E1α1 (PDHA1), a critical component of the PDH complex [46]. p53 also promotes mitochondrial OXPHOS through the upregulation of apoptosis-inducing factor (AIF) and synthesis of cytochrome c oxidase 2 (SCO2). AIF, a mitochondrial flavoprotein, is essential for the proper assembly and functionality of mitochondrial respiratory complex I, while SCO2 is required for the assembly of mitochondrial DNA-encoded COX II subunit into complex IV [47,48] (Figure 3). Interestingly, in fully senescent cells, p53 levels decline, contributing to a metabolic shift from OXPHOS to glycolysis. This transition is characterized by elevated levels of glycolytic and PPP metabolites and transcripts, along with reduced TCA cycle metabolites [41].

Among the metabolites of the ESM, citrate is particularly noteworthy due to its involvement not only in aging, but also in other physiological and pathological processes, including caloric restriction, type 2 diabetes, blood pressure regulation, adipocyte inflammation, epigenetic regulation, and cancer [28]. Senescent cells accumulate citrate extracellularly, with levels more than 20-fold higher outside the cells compared to the interior or controls. This export aligns with the incompatibility of high intracellular citrate concentrations with glycolysis [49]. Citrate is generated during the initial step of the TCA cycle within the mitochondrial matrix, and its transport to the cytosol is mediated by the mitochondrial citrate carrier (CIC) (Figure 3). Since citrate is involved in different metabolic pathways, its flux between mitochondria and cytosol has been associated with metabolic flexibility in both yeast models and cancer cells [50,51]. Consequently, alterations in the expression or activity of CIC have been linked to many pathological conditions [52]. The cytosolic citrate pool is also influenced by plasma membrane citrate transporters, such as *SLC13A5*/INDY and plasma membrane CIC (pmCIC), a bidirectional transporter derived from an alternative splice variant of *SLC25A1*, the gene encoding the mitochondrial CIC. While CIC predominantly resides in mitochondria, pmCIC localizes to the plasma membrane, where it functions as a citrate exporter under normal physiological conditions [53]. Although the citrate transporters involved are well-identified, it remains unclear whether the extracellular citrate accumulation observed during senescence results from increased export or decreased import [28].

## 4. The Crosstalk Between Telomere Maintenance and Mitochondrial Functionality in Different Cell Types

The molecular mechanisms governing the interplay between telomeres and mitochondria have been extensively studied in fibroblasts, yet they remain poorly understood in other cell types, such as immune and cancer cells. Unravelling the mediators of this crosstalk and their specific roles could provide valuable insights into the molecular basis of immune cell dysfunction, as well as the mechanisms driving tumorigenesis. The latest findings are summarized in Table 1 and analyzed in the following paragraphs.

### 4.1. Crosstalk Between Telomeres and Mitochondria in Fibroblasts

In fibroblasts, hTERT overexpression protects mitochondria from oxidative damage. However, significant mitochondrial defects have been associated with reduced telomere length and genomic instability. Conversely, telomere attrition and dysfunction activate a DDR and disrupt NAD metabolism along with related pathways, further contributing to mitochondrial abnormalities [35].

In human fibroblasts, oxidative stress induces the overexpression of hTERT through the SIRT1/FOXO3a/c-MYC pathway, reducing mtDNA damage [59]. This protective effect does not appear to stem from enhanced mtDNA repair mechanisms. Instead, hTERT overexpression increases the levels of manganese superoxide dismutase (MnSOD) and FOXO3a proteins, enhancing the cellular antioxidant defense system. It means that telomerase mitigates mtDNA damage by strengthening antioxidant defenses rather than directly influencing DNA repair processes [54].

However, in the presence of OXPHOS defects, which cause a metabolic shift toward glycolytic ATP production, accelerated telomere shortening and epigenetic aging have been observed. This link between OXPHOS dysfunction and telomere attrition may occur disproportionately to or independently of cell division, as evidenced by hypermetabolic fibroblasts showing a disconnection between telomeric repeat loss and genome replication [55]. Indeed, even with an adaptive transcriptional upregulation of telomere protection complex components, OXPHOS defects significantly increase the rate of telomere erosion per cell division [55]. Moreover, even mild alterations in OXPHOS function, potentially caused by common genetic variants in complex I subunit genes, can influence lifespan and cellular aging processes [60].

Additionally, in human primary fibroblasts, the appearance of chromosome breaks has been associated with dysfunction in the mitochondrial CIC, strongly indicating that the correct transport of citrate between mitochondria and cytosol is required for genome stability [61]. Indeed, citrate exported from mitochondria by CIC serves as a substrate for ATP citrate lyase, which produces acetyl-CoA that is essential not only for lipid anabolism and cell growth, but also for histone acetylation [62].

Short telomeres activate p53, reducing SIRT1 expression and deacetylation activity, which adversely affects mitochondrial function. Concurrently, telomere dysfunction-induced DDR increases cluster of differentiation 38 (CD38) activity, a major NAD-consuming enzyme, leading to NAD depletion and limiting its availability for SIRT enzymatic activity. This results in reduced SIRT1 activity, lower PGC-1α expression, and impaired mitochondrial biogenesis [35]. Restoring NAD levels has been shown to improve mitochondrial function in a SIRT1-dependent manner [63]. Additionally, NAD depletion impairs mitochondrial quality control by disrupting SIRT1-mediated mitophagy, which is critical for removing damaged mitochondria [64]. These disruptions contribute to mitochondrial abnormalities, including structural defects and functional decline [35].

### 4.2. Crosstalk Between Telomeres and Mitochondria in Immune Cells

T cells are critical for protecting the body against infections and malignancies; however, the factors driving their senescence are not yet fully understood. T cells senescence is closely associated with diminished proliferative capacity and responsiveness to antigens, which correlate with telomere shortening and mitochondrial dysfunction [65]. Experimental studies in Jurkat T cells have revealed a bidirectional relationship between telomeres and mitochondria: oxidative damage targeted to telomeres not only accelerates telomere erosion but also compromises mitochondrial function, while damage directed at mitochondria induces mitochondrial dysfunction alongside telomere attrition. These interconnected impairments exacerbate T cell senescence, impair functionality, and promote apoptosis [56].

Mitochondrial dysfunction increases ROS production, leading to telomeric DNA damage due to telomeres’ high sensitivity to ROS-induced genotoxicity [29]. When ROS levels surpass the cell’s scavenging or antioxidant capacity, oxidative stress accumulates, causing telomere damage and accelerated shortening. Conversely, mitigating oxidative stress with antioxidants slows telomere shortening and extends replicative lifespan [56]. Oxidative stress notably results in a significant increase in TIFs, accompanied by a reduction in telomeric DNA content. The base excision repair (BER) pathway, crucial for repairing oxidized DNA bases, plays a central role in countering telomeric DNA loss. However, elevated oxidative stress is associated with reduced expression of BER-related DNA repair enzymes, linking this pathway to oxidative stress-induced telomere attrition [56].

On the other hand, recent research highlights the role of telomere integrity disruption in inducing T cell senescence and apoptosis, as shown by using KML001, a telomere-targeting drug. KML001 disrupts telomeric integrity, triggering a telomeric DDR that results in mitochondrial dysfunction. Specifically, KML001 treatment leads to increased mitochondrial swelling, reduced mitochondrial membrane potential, and impaired mitochondrial function, including decreased OXPHOS, mtDNA content, oxygen consumption, and ATP production. Mechanistically, KML001-induced telomeric DDR activates the p53 signaling pathway, which represses the expression of PGC-1α. PGC-1α controls transcription factors such as NRF-1 and estrogen-related receptor alpha (ERRα). These factors govern the expression of essential metabolic genes involved in cellular growth, mitochondrial respiration, heme biosynthesis, and mitochondrial DNA transcription and replication [29]. Consequently, p53-mediated repression of PGC-1α disrupts mitochondrial homeostasis in T cells, contributing to senescence and functional decline [29,30]. Targeting this pathway could provide novel strategies to mitigate telomere damage-induced mitochondrial dysfunction and T cell aging, potentially addressing immune aging-related diseases and improving immune system resilience.

### 4.3. Crosstalk Between Telomeres and Mitochondria in Cancer Cells

Cancer cells exhibit hallmark characteristics such as replicative immortality and metabolic flexibility, underscoring the interplay between telomeres and mitochondria [66]. A well-documented metabolic feature of cancer cells is the Warburg effect, characterized by an elevated glycolytic rate even in the presence of adequate oxygen levels, coupled with reduced mitochondrial activity [67]. Simultaneously, glucose metabolism participates in telomere maintenance by telomerase regulation. High glucose levels have been shown to epigenetically modulate TERT expression, increasing its transcription and promoting tumor proliferation by acetylating histone H3K9 on the TERT promoter [68]. Then, TERT modulates glycolysis by regulating key enzymes like HK2, thereby promoting cellular energy production in cancer cells [69]. It means that TERT plays a pivotal role in cancer progression and is intricately linked to glucose metabolism.

Thus, marked alterations in mitochondrial function and TERT upregulation are critical for enabling the metabolic flexibility that sustains cancer cell growth and survival. Despite the decreased reliance on mitochondria as a major energy source for cancer cells, genetic studies indicate a continued vital role in the maintenance of mitochondrial function in cancer. The need to maintain mitochondrial function highlights the importance of keeping intracellular ROS levels within tolerable limits, especially considering the high sensitivity of telomeres to oxidative stress [70].

A decline in mitophagy has been shown to promote tumorigenesis by causing the accumulation of damaged mitochondria, leading to increased ROS generation. This, in turn, directly induces DNA double-strand breaks in telomeres [57]. Conversely, telomere shortening appears to negatively affect mtDNA copy number (mtDNAcn) by suppressing the expression of genes involved in mtDNA replication, such as NRF1 [58].

Identifying more mediators between mitochondria and telomeres is pivotal for understanding the underlying molecular basis, especially in the context of tumorigenesis. Both cytosolic and mitochondrial enzymes have been recognized as key players linking cellular metabolism to telomere stability [71,72]. For example, mitochondrial methylcrotonoyl-CoA carboxylase (MCCC2), which participates in leucine and isovaleric acid catabolism, supports both mitochondrial dynamics and telomere integrity, although the underlying mechanisms remain to be fully elucidated [71]. The glycolytic enzyme glyceraldehyde 3-phosphate dehydrogenase (GAPDH), instead, directly binds to TERC and inhibits telomerase activity, leading to telomere shortening and senescence. This interaction between GAPDH and telomerase is negatively regulated by the GAPDH substrates NAD and glyceraldehyde 3-phosphate, indicating that these substrates play an important role in preventing GAPDH from switching from the metabolic pathway to telomere signaling [72].

## 5. The Peculiar Case of Sperm Cells

Sperm cells exhibit numerous distinctive features compared to somatic cells, including unique characteristics of both their mitochondria and telomeres.

Sperm mitochondria, just like somatic mitochondria, are considered the powerhouse of the cell. Still, they differ structurally and functionally from somatic ones, being confined to the beginning of the flagellum (midpiece) and tightly wrapped around the axoneme. Furthermore, sperm mitochondria show an elongated form, are strongly linked to each other and possess specific isoforms of protein and isoenzymes. In sperm cells, mitochondrial activity is crucial for hyperactivated motility and capacitation, with the latter involving a substantial increase in mitochondrial functionality [73]. Exogenous nutrients reach mitochondria via membrane carrier proteins, with CIC playing a key role in sperm metabolism, especially during the early stages of capacitation [74,75]. CIC transports citrate from mitochondria to cytosol, where it provides acetyl units for cholesterol biosynthesis, essential for modulation of sperm membrane fluidity, and generates nicotinamide adenine dinucleotide phosphate (NADPH) molecules required for both cholesterol biosynthesis and NADPH oxidase activity [74].

In human sperm, each mitochondrion contains, on average, one copy of the mitochondrial genome [76]. mtDNA encodes, in addition to transfer RNA (tRNAs) and ribosomal RNA (rRNAs), 13 essential proteins for respiration and energy production, and fluctuations in mtDNAcn can affect mtDNA gene expression and mitochondrial respiration. The molecules of sperm mtDNA are very few (1–10^3^ copies) as compared with mtDNA copies in somatic cells (10^2^–10^4^ copies) [73], and this difference in content could depend on the small number of mitochondria retained within the sperm cell (roughly 50-75 mitochondria) [77]. Most mitochondria are, indeed, eliminated during spermatogenesis to ensure the matrilinear inheritance of these organelles [78].

Additionally, the lack of histones and the mtDNA repairing activity that is less efficient in sperm than in somatic cells makes the sperm mitochondrial genome particularly susceptible to mutations and deletions [76], compromising its stability and integrity. Maintaining mtDNA stability is hence essential for mitochondrial function and cellular homeostasis [79]. Therefore, quantitative and qualitative mtDNA assays are necessary to evaluate sperm functionality and male fertility [80,81]. Evidence suggests that impaired sperm functionality is associated with reduced mtDNA integrity and increased mtDNAcn [82,83]. However, the increase in mtDNAcn may represent a compensatory mechanism to mitigate mitochondrial dysfunction [84].

Telomeres of sperm cells, as well as sperm mitochondria, differ substantially from the telomeres of somatic cells. It has been established that telomere length in human spermatozoa ranges between approximately 6–20 kb and is significantly longer than in somatic cells [85,86,87]. In contrast to most cell lines and tissues, telomerase, responsible for maintaining telomere length, is more active in sperm cells. Furthermore, mature spermatozoa show telomeres longer than their precursor cells despite the progressive reduction in telomerase activity [88,89,90,91]. Unlike somatic cells, telomere length in sperm increases with age [92], and the offspring of older fathers at conception show longer telomeres than younger fathers’ offspring [93]. Additionally, mild oxidative stress conditions have been shown to induce telomere lengthening in sperm cells [94]. The effect of oxidative stress on sperm telomere length (STL) may depend on the timing of oxidative insults. Oxidative stress occurring in premeiotic germ cells can trigger an adaptive response, upregulating telomerase and lengthening telomeres. However, oxidative stress affecting post-meiotic germ cells or mature spermatozoa, which lack effective DNA repair and telomerase activity, shortens telomeres. Thus, both the intensity and timing of oxidative stress are key factors in determining the telomere response [95].

Studying reproductive damage caused by environmental pollutants can advance understandings of the molecular links between mitochondria and telomeres in germ cells [96]. For instance, exposure to benzo[a]pyrene (BaP), a polycyclic aromatic hydrocarbon, has been shown to inhibit TERT transcription via the SIRT1/FOXO3a/c-MYC pathway. This results in telomere dysfunction and mitochondrial damage, specifically impairing complex I activity and subunit expression in male rat spermatogenic cells [97].

Given that telomeres are histone-bound and positioned near nuclear boundaries, this structural characteristic is hypothesized to influence the fertilization process [98]. Therefore, telomere maintenance is crucial for spermatogenesis and conception [99]. Many studies have highlighted a correlation between short STL and reduced sperm quality; precisely, oligozoospermic subjects have STL shorter than normozoospermic subjects [100]. Moreover, men with normal semen parameters but with short STL show increased DNA fragmentation and reduced protamination compared to normozoospermic men with long STL [101,102]. Based on this evidence, STL has been suggested as a new reliable biomarker of sperm fertilization capacity and embryo quality [103,104,105].

In addition to the gradual silencing of telomerase, several endogenous and exogenous factors, including ROS, can shorten STL [106]. Usually, in sperm cells, a delicate balance between ROS production and antioxidant activity is maintained to guarantee the correct sperm functionality [107]. In sperm, ROS are produced mainly from complexes I and III, unlike somatic mitochondria [108]. Even tough ROS are essential for physiological sperm functions such as capacitation, hyperactivation, acrosome reaction, and egg fertilization; however, it has been observed that an excess of oxidative stress negatively correlates with STL [94]. The main endogenous source of increased ROS is mitochondrial dysfunction that can be due to alterations in OXPHOS system and genetic variations in mtDNA (such as mutations, deletions, and copy number) [55].

Although it is well-known that telomere length is ROS-sensitive, research on the potential correlation between telomeres and mitochondrial features in human sperm is still limited [109,110]. Significant positive correlations between STL and both sperm nuclear DNA damage and sperm mtDNAcn, and a significant negative correlation between STL and sperm mtDNA integrity have been observed in healthy young men [111]. The positive correlation between STL and mtDNAcn suggests a potential compensatory mechanism linking telomere elongation to reduced sperm functionality [111]. Moreover, a positive association between STL and DNA fragmentation index (DFI) has been reported in healthy young men, possibly due to telomerase activation by mild oxidative stress, which can simultaneously induce genomic damage and STL elongation [111].

## 6. Conclusive Remarks and Future Directions

Telomeres, the protective caps at the ends of eukaryotic chromosomes, play a critical role in maintaining genomic stability, but progressively shorten with cell divisions due to replication constraints and oxidative stress primarily driven by mitochondrial ROS. This creates a reciprocal relationship between telomere attrition and mitochondrial dysfunction, with p53 and PGC coactivators serving as key regulators [27]. Mild oxidative stress can trigger telomerase overexpression, which protects mitochondria from damage. However, in the presence of OXPHOS defects or during senescence, mitochondrial function declines, leading to increased ROS levels that cause telomere shortening, chromosomal fusions, and activation of the DDR [17,18,19]. Conversely, telomeric DNA damage triggers DDR, activating p53, suppressing PGC-1α and PGC-1β, and reducing mitochondrial biogenesis [4,29,35]. This dual effect of mitochondrial ROS generation on telomere maintenance requires further exploration, as it has been studied in both fibroblasts and immune cells [35,54,55]. Telomere dysfunction also reprograms cellular metabolism, shifting energy production from OXPHOS to glycolysis [28,41]. This metabolic reprogramming contributes to the accumulation of TCA cycle metabolites, particularly citrate, linking telomere damage to broader metabolic alterations. Notably, marked alterations in mitochondrial function and TERT upregulation are critical characteristics of cancer cells. Approximately 15% of human cancers maintain telomeres in the absence of telomerase activity through an alternative lengthening of telomeres (ALT) mechanism and exhibit amplification or overexpression of PGC-1β [112].

Telomere maintenance and mitochondrial metabolism are also fundamental to sperm fertilizing ability, but many unanswered questions remain about the connection between mitochondria and telomeres in sperm cells. For instance: Can specific metabolic pathways influence telomere stability and genome integrity? Is telomere maintenance linked to the expression of mitochondrial proteins? Could abnormalities in the expression or activity of the mitochondrial CIC impact telomere stability? Sperm cells can use citrate as an energy source under low-sugar conditions, but the role of CIC in sperm quality assessment remains unclear. A potential correlation may exist between CIC levels and sperm DNA stability, with altered CIC expression possibly linked to abnormal semen parameters. Further research is needed to address these questions and clarify the role of CIC in modulating DNA stability, STL, and overall sperm quality.

## Figures and Tables

**Table 1:** Key cause–effect relationships linking telomere maintenance and mitochondrial function in different cell types.

Cell Type	Cause	Effect	Refs.
Fibroblasts	Oxidative stress	Overexpression of telomerase	[54]
	OXPHOS defects	Accelerated telomere shortening	[55]
	Telomere shortening	Impaired mitochondrial biogenesis	[35]
Immune cells	Oxidative stress	Increase in TIFs and telomere attrition	[56]
	Disrupted telomeric integrity	Mitochondrial swelling with decreased membrane potential, OXPHOS function, and mtDNA content	[29]
Cancer cells	Decline in mitophagy	DNA double-strand breaks in telomeres	[57]
	Telomere shortening	Reduced mtDNA replication	[58]

## Data Availability

Not applicable.

## Abbreviations

The following abbreviations are used in this manuscript:

6-PGL	6-phosphogluconolactone
8-OHdG	8-hydroxy-2′-deoxyguanosine
ADP	adenosine diphosphate
AIF	apoptosis-inducing factor
ALT	alternative lengthening of telomeres
ATP	adenosine triphosphate
BaP	benzo[a]pyrene
BER	base excision repair
CD38	cluster of differentiation 38
CIC	mitochondrial citrate carrier
c-MYC	cellular myelocytomatosis oncogene
DDR	DNA damage response
DFI	DNA fragmentation index
Drp1	dynamin-related protein 1
E-box	enhancer box
ERRα	estrogen-related receptor alpha
ESM	extracellular senescence metabolome
F-1,6-BP	fructose 1,6-bisphosphate
F-2,6-BP	fructose 2,6-bisphosphate
F-6-P	fructose-6-phosphate
FADH2	flavin adenine dinucleotide
Fis1	mitochondrial fission 1
FOXO3a	forkhead-box-protein O3
G-6-P	glucose-6-phosphate
G6PDH	glucose-6-phosphate dehydrogenase
GAPDH	glyceraldehyde 3-phosphate dehydrogenase
GLUT	glucose transporter
H2AX	H2A histone family member X
HK2	hexokinase 2
HnRNPA	heterogeneous nuclear ribonucleoproteins
HIF-1α	hypoxia-inducible factor-1α
hTERT	human TERT
MCCC2	mitochondrial methylcrotonoyl-CoA carboxylase
Mff	mitochondrial fission factor
Mfn	mitofusin
MiD49/51	mitochondrial dynamics proteins of 49 and 51 kDa
MnSOD	manganese superoxide dismutase
mtDNA	mitochondrial DNA
mtDNAcn	mtDNA copy number
NAD	nicotinamide adenine dinucleotide
NADPH	nicotinamide adenine dinucleotide phosphate
NRF	nuclear transcriptional respiratory factor
OPA1	optic atrophy 1
OXPHOS	oxidative phosphorylation
PDH	pyruvate dehydrogenase
PDK2	pyruvate dehydrogenase kinase 2
PFK1	phosphofructokinase 1
PGC	peroxisome proliferator-activated receptor-γ coactivator
pmCIC	plasma membrane CIC
POT1	protection of telomeres protein 1
PPP	pentose phosphate pathway
RAP1	repressor activator protein 1
rRNA	ribosomal RNA
ROS	reactive oxygen species
SCO2	synthesis of cytochrome c oxidase 2
SIRT	sirtuin
STL	sperm telomere length
TCA	tricarboxylic acid
TERC	telomerase RNA component
TERRA	telomeric repeat-containing RNA
TERT	telomerase reverse transcriptase
TFAM	mitochondrial transcription factor A
TIFs	telomere dysfunction-induced foci
TIGAR	TP53-inducible glycolysis and apoptosis regulator
TIN2	TERF1-interacting nuclear factor 2
TPP1	adrenocortical dysplasia protein homolog
TRF	telomeric repeat binding factor
tRNA	transfer RNA
